# Experimental Determination of the Coefficient of Restitution for Selected Modern Hybrid Composites

**DOI:** 10.3390/ma14195638

**Published:** 2021-09-28

**Authors:** Witold Wojciech Skórski, Marcin Obszański, Maciej Zawisza

**Affiliations:** 1 Faculty of Power and Aeronautical Engineering, Institute of Aeronautics and Applied Mechanics, Warsaw University of Technology, ul. Nowowiejska 24, 00-665 Warsaw, Poland; wskor@meil.pw.edu.pl (W.W.S.); mobszan@meil.pw.edu.pl (M.O.); 2 Faculty of Automotive and Construction Machinery Engineering, Institute of Machine Design Fundamentals, Warsaw University of Technology, ul. Narbutta 84, 02-524 Warsaw, Poland

**Keywords:** modern hybrid composites, measured and computational coefficient of restitution

## Abstract

Composite materials (fiber reinforced plastics, FRPs) are successfully utilized in the production of various mechanical devices, including land vehicles, marine vessels, and aircrafts. They are primarily used for the production of body parts and hulls. Due to their importance, certain requirements relating to the mechanical properties of the materials used must be met for such applications. One aspect of the passive safety of vehicles is the effects of a possible collision with another object. The behavior of the structure in such a case can be determined based on the coefficient of restitution, which is a measure of energy dissipation after an impact. This paper presents the results of measuring the value of the coefficient of restitution for the selected composite materials, utilizing various reinforcement materials including different types of fibers and wooden veneer. The selected materials included glass, carbon, Kevlar fibers, and veneer from exotic wood in an epoxy resin matrix. The tested samples were made using various methods in order to understand the influence of the technology on the value of the coefficient. The authors determined the coefficient values utilizing two methods based on the measurement of two different physical quantities. In the first case, the height of the rebound of the ram was measured using a fast digital camera; in the second case, the time between successive rebounds of the tool was measured, determined based on the signal from the acceleration sensor. The authors compared the results of the coefficient values obtained using these methods and examined the relationship between the rebound energy and the value of the coefficient of restitution. The results have been discussed, and some conclusions have been made. Among other things, it seems that both methods of measurement are interchangeable with regard to lower impact velocities corresponding to lower heights (up to 300 mm) of the drop of the ram used in the tests.

## 1. Introduction

At present, various types of composites are replacing widely used traditional materials. Future solutions revolve around a type of technology that allows for the production of hybrid structures based on carbon fibers, glass fibers, aramid fibers, and engineered wood. An important problem to resolve is the difficulty in determining their mutual relations and the mechanical properties of the final product [1,2,3,4,5]. One cannot talk here about additive properties of materials, because the composite that they are made up of acquires material properties only after the shaping of the laminate. So-called “dedicated materials”, with a specific structure that depends on the type of acting loads, are increasingly being used. The spectrum of their application is wide ranging, from aviation to automotive, and marine to space industries [6,7,8,9]. Additionally, the military industry is looking for materials with specific properties. Therefore, it is extremely important to determine the strength of these materials, both for static and dynamic loads, including fatigue loads [10,11,12,13,14,15]. Aside from the traditionally determined parameters for construction materials, such as Young’s modulus, Poisson’s ratio or fatigue limits, it is important to determine other parameters related, for example, to the dissipation of deformation energy and vibration damping. It is important, among other things, to determine the coefficient of restitution for newly produced materials. The coefficient of restitution, ε, is one of the parameters relating to the physical properties of a material, and to its response to excitation in the form of dynamic point impact forces [16,17,18]. Most of the hypotheses on energy dissipation during the collision of two bodies presented in the literature [19] focus on the law of conservation of energy.

We consider the collision process in two time periods. During the first phase, there is an increase in the value of a momentary force during the contact of two bodies and an associated increase in deformations. Deformations can be divided into local deformations occurring at the point of contact at the initial moment, and general deformations extending beyond the contact area. The (time) limit of the first period is determined by the value of the maximum contact force, which is at the same time associated with the creation of maximum local deformations. In the second phase, the value of the contact force decreases to zero and the local and total deformations disappear (of course, provided that the boundary values of the material’s strain are not exceeded). Overall, the ratio of these two phases can be considered as a measure of the coefficient of restitution. The coefficient of restitution, ε, introduced by Newton, is determined by the ratio of the impulse of a momentary force in the second phase to the impulse of a momentary force during the first phase. In other words, it is concerned with determining which part of the impulse of the first phase of the collision will be recovered during the second phase. The consequence of the impact is the propagation of vibrations, which in the case of complex composite hybrid structures is a critical phenomenon due to complex (non-linear) damping. Therefore, it is necessary to analyze the impact energy absorption process.

## 2. Materials

Due to material costs, manufacturing costs, and the construction of a specialized test stand, experiments with the use of full-sized objects are very expensive. Because of cost, the tests were carried out using standard samples of small dimensions.

**Figure 1:**
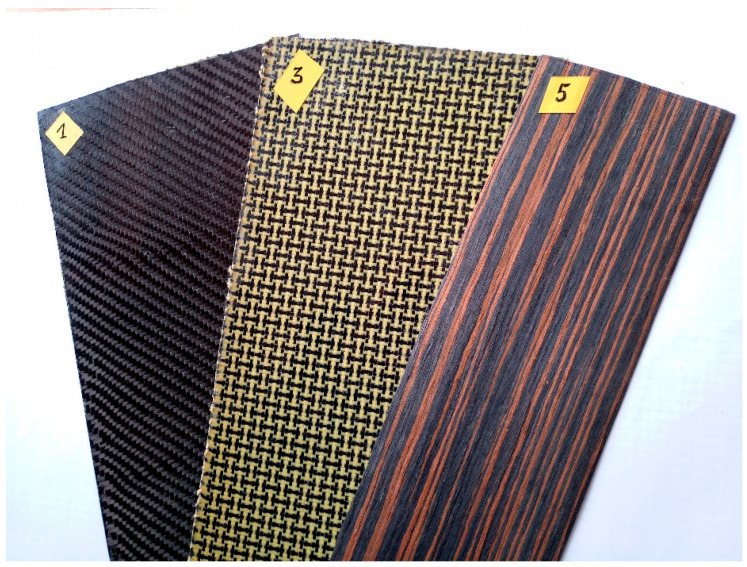
Examples of tested samples: 1—1P/1BP (carbon side exposed), 3—3P/3BP (Kevlar–carbon side exposed), 5—5P/5BP (veneer side exposed).

Several dozen material samples were made. All of them were squares with a side length 120 mm. They differed in the material of reinforcement, matrix, layer numbers, and setup. Examples of material samples are shown in Figure 1. All of the materials were tested to determine their properties, but for the purpose of this paper only five representative types were selected. To determine the impact of the manufacturing method on the final mechanical properties (including the coefficient of restitution), two methods were used to prepare samples: manual wet lay-up with a vacuum bag for the curing time and manual wet lay-up without a vacuum bag. The layers’ setup and the main physical properties of these materials are shown in Table 1. Table 2 contains basic information (i.e., fabric type and grammage) on the reinforcement materials used.

## 3. Test Stand and Course of the Experiment

**Figure 2:**
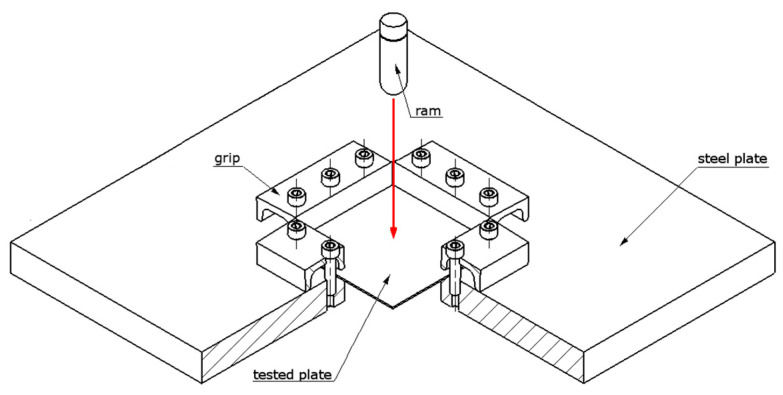
Test stand used in the described study.

The described tests were carried out on the test stand shown in Figure 2. It consisted of:

- A ram equipped with an acceleration sensor;
- A guide tube for the ram during impact (not included in the figure);
- A holder with the tube attached (not included in the figure);
- A steel plate with a square cutout for fixing specimens.

**Figure 3:**
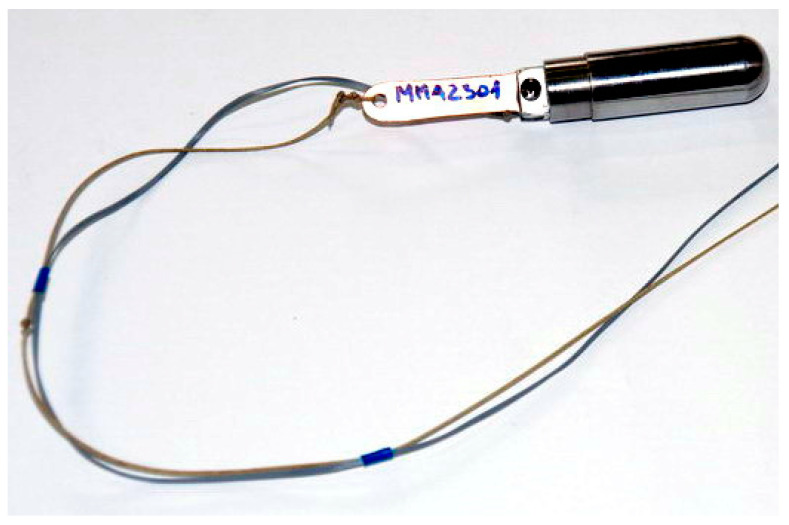
Ram used during the tests with an acceleration sensor attached to the rear.

The ram (Figure 3) used in the tests was in the form of a steel cylinder crowned with a hemisphere. The outer diameter of the ram in its front spherical end was 20 mm. A microelectromechanical acceleration sensor (MEMS) (Figure 3) was mounted on the rear of the ram. The weight of the ram with the sensor attached was 149.1 g.

The ram was placed in a polycarbonate tube, which acted as a guide during impacts. This was to ensure that impacts occurred along a vertical line without the reaction of tangential forces. The inner diameter of the tube was 26 mm. During the tests, the samples were attached to a steel plate. A square-shaped cutout with a side length of 100 mm was made in the central part of the plate. At the edges of the cutout, four grips were attached to ensure adequate pressure.

**Figure 4:**
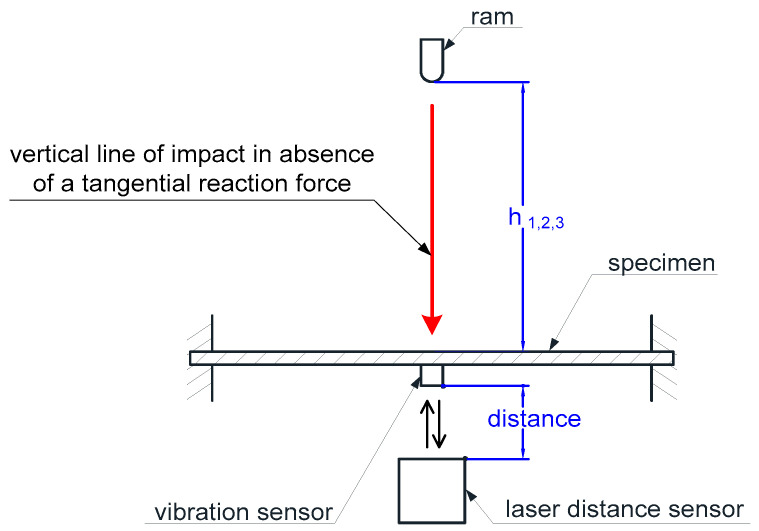
Mounting of the test plate.

Linear gauges were attached to the guide, which were used to determine the height of the ram’s rebound during the analysis of the film recording of the tests. A high-speed digital camera was used to make the recording. Laser distance sensors were positioned under the cutout, below the base. A record from the laser displacement sensors (Figure 4) was used to analyze the plate vibrations after the ram’s impact. Depending on the type/kind of conducted study, one or two sensors were used. The first one was positioned so that the laser beam was directed towards the center of the plate, i.e., the place opposite the point of impact of the ram, and the second one measured the deflection of the plate closer to its edge, at some distance from the point of impact, which enabled the identification of wave propagation (transmittance).

The following values were measured during the tests:

- Acceleration by means of a sensor attached to the ram;
- Plate deflection by means of the distance sensors;
- The height of a rebound;
- Time between successive rebounds of the ram identified on the basis of the analysis of film frames.

The height of the ram’s rebound after successive impacts on the plate was determined based on an analysis of the recorded film.

It should be emphasized that determining the value of the coefficient of restitution must be carried out in conditions not exceeding the threshold of damage to the structure of the tested material. Each sample’s damage limit was determined in separate tests.

The experiment was performed by measuring the described parameters during the drop of the ram from heights of 100, 200, 300, 350, 400, 450, and 500 mm. Due to technical reasons, the height of the rebound was not recorded when the ram was dropped above 300 mm.

## 4. Results and Discussion

**Figure 5:**
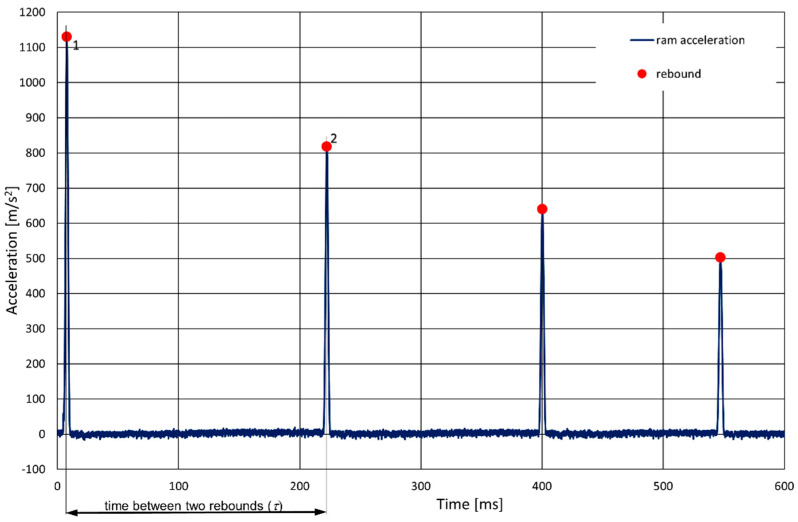
Fragment of the record of acceleration acting on the ram (1BP sample, drop from a height of 100 mm). Time between the first and second rebound is marked [9].

The acceleration acting on the ram, noted during the tests, was used to determine the coefficients of restitution of the tested plates (Figure 5). Each impact on the plate is clearly visible in the record of the ram’s acceleration. The successive peaks observed in the graphs correspond to successive impacts. On this basis, it was possible to determine the time between successive impacts of the ram.

The coefficient of restitution is found using the following formula [19]:

(1) $$\varepsilon =\frac{\Delta w^{\prime }}{\Delta w}$$

where:

Δ*w*′—relative velocity of the bodies after the collision,
Δ*w*—relative velocity of the bodies before the collision.

The plate to which the samples were attached had a mass many times greater than the ram and was fixed. Therefore, in the analyzed case, Formula (1) can be transformed into the following form by specifying the calculated coefficient as the measurement coefficient of restitution:

(2) $$\varepsilon_{pom}=\sqrt{\frac{{h^{\prime }}_{pom}}{h_{pom}}}$$

where:

${h^{\prime }}_{pom}$—height reached by the ram after a rebound,
$h_{pom}$—ram’s drop height.

Assuming that the ram’s energy during the test dissipated only at the moment it hit the plate, it is possible, on the basis of the time between individual impacts, to determine the height that the ram reached after an impact using the following dependence:

(3) $$h_{obl}=\frac{1}{2}g{\left(\frac{\tau }{2}\right)}^{2}=\frac{1}{8}g\tau^{2}$$

where:

*g*—standard gravity,
*τ*—time between rebounds.

Hence, by inserting the heights of the rebounds from Formula (3) into Formula (2), the values of the analytical coefficient of restitution for successive rebounds, hereafter referred to as the computational coefficient of restitution, were determined.

**Figure 6:**
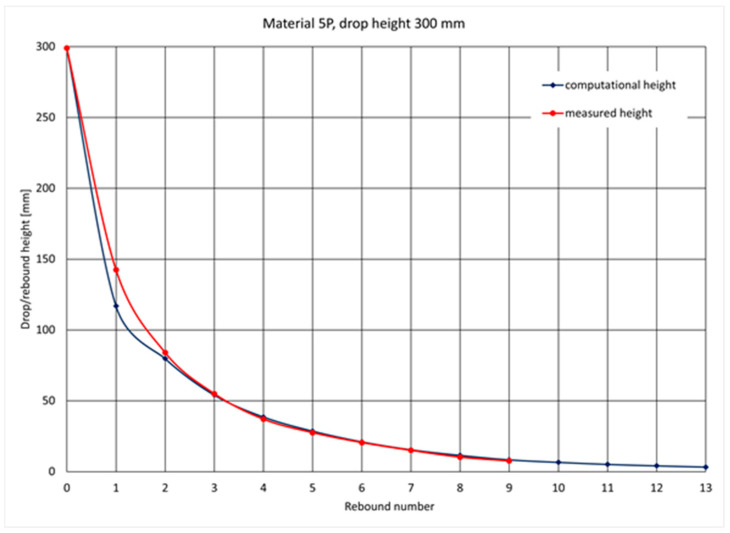
Example of determining the height of the ram’s rebound by computational and measurement methods.

**Figure 7:**
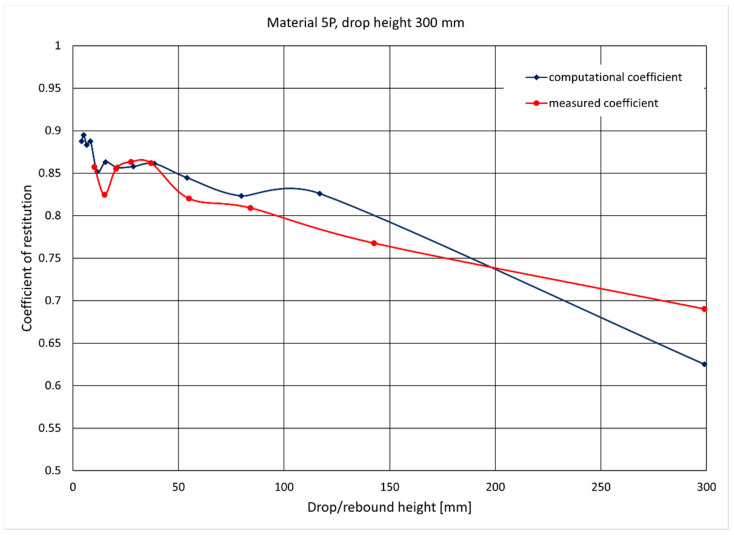
Record of changes in the computational and measurement coefficients of restitution during a drop from a height of 300 mm. Successive values marked on the graph correspond to rebounds from the plate.

Figure 6 shows a comparison of the measurement results and the calculations of the height of the ram’s rebound for a given sample, and Figure 7 shows the values of the computational and measurement coefficients of restitution for the same sample.

(4) $$\varepsilon_{obl}=\sqrt{\frac{{h^{\prime }}_{obl}}{h_{obl}}}$$

**Figure 8:**
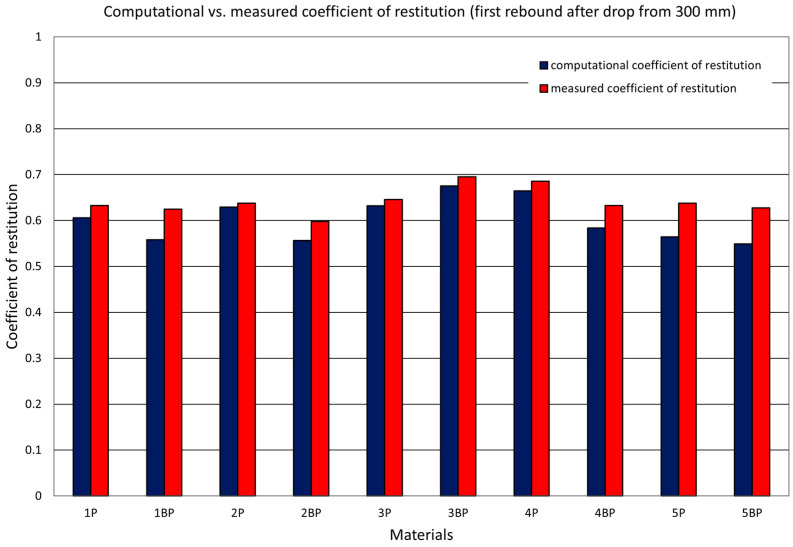
Coefficients of restitution. Comparison of the values of computational and measurement coefficients. Drop from a height of 300 mm.

**Figure 9:**
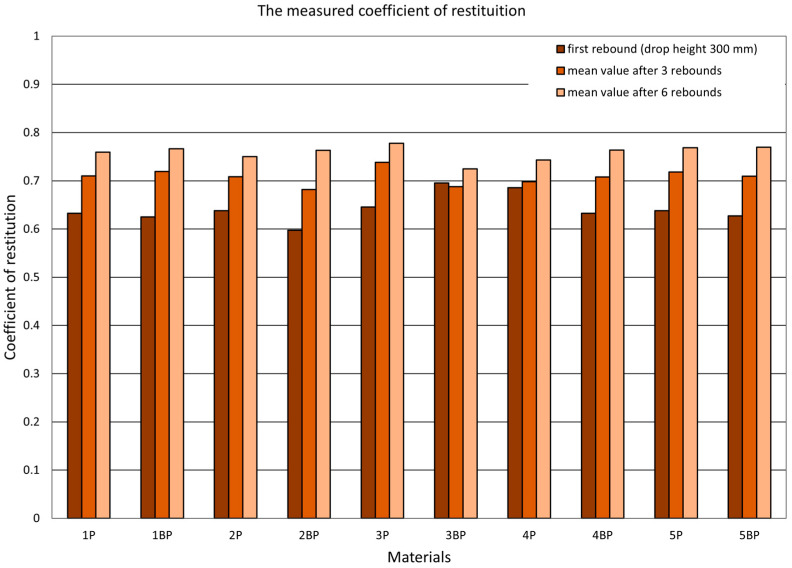
Measurement coefficient of restitution. Comparison of the coefficient values for the first rebound (drop height of 300 mm) and average values for the first three and six rebounds.

**Figure 10:**
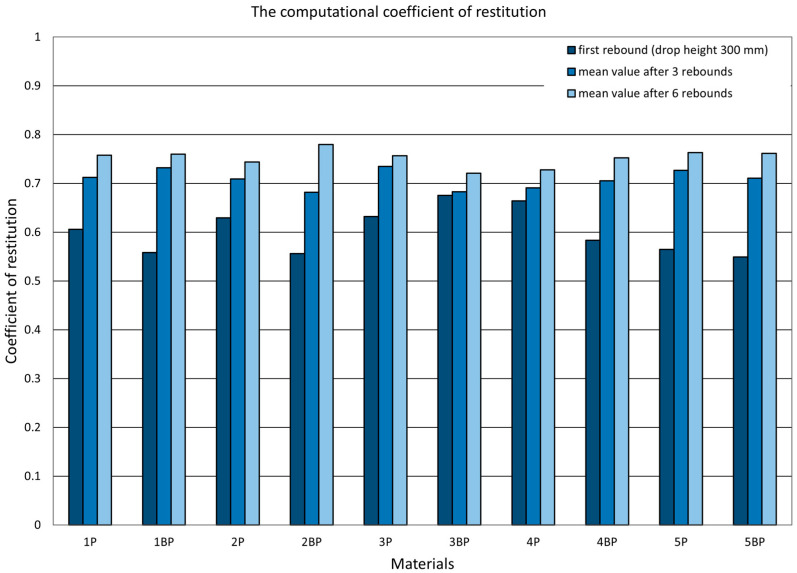
Computational coefficient of restitution. Comparison of the values for the first rebound and average values after the first three and six rebounds.

Alongside determining the value of the coefficient of restitution using the two described methods, the authors investigated the dependence of the value of the coefficient of restitution on the initial drop height by considering the successive bounces of the ram. In Figure 8, Figure 9 and Figure 10, there are graphs displaying the values of the coefficients of restitution determined for the plates. Figure 8 shows a comparison of the computational coefficient of restitution for the height of the first drop and the measurement coefficient of restitution based on an analysis of the time between the first and second rebounds. Figure 9 presents a comparison of the measurement coefficient of restitution for the first rebound from a drop height of 300 mm and the average values calculated after three and six rebounds, while Figure 10 shows a comparison of the value of the computational coefficient of restitution for the conditions shown in Figure 9.

Further research was carried out in order to determine whether the value of the coefficient of restitution depends on the height of a drop, i.e., on the energy with which the ram hits the plate. The ram fell freely from a height of 100, 200, and 300 mm. During the tests, the ram acceleration and plate deflection were measured at two points—in the middle of the plate and near the edge. There was also a film recording of the tests. The values of the computational coefficient were determined on the basis of the ram acceleration record. The values of the measurement coefficient were determined on the basis of the analysis of video recordings. The following materials were used in the study: 1P, 1BP, 2P, 3P, 4P, 4BP, and 5P.

In order to describe the dependence of the coefficient of restitution on the impact energy, the computational and measurement values obtained during the study were used to determine the linear regression function, in accordance with the following formula:

(5) ε(h)=ah+b

where:

*a*—slope of the linear approximation of the dependence *ε*(*h*),
*b*—intercept of the linear approximation of the dependence *ε*(*h*).

Table 3 presents the parameters of the linear regression of the coefficient of restitution for the materials used in the study. It also includes correlation coefficients of the approximation function, and the values obtained in the experiment as well as the standard deviation of regression calculated for the entire set of measurement points after removing values considered to be gross errors.

**Figure 11:**
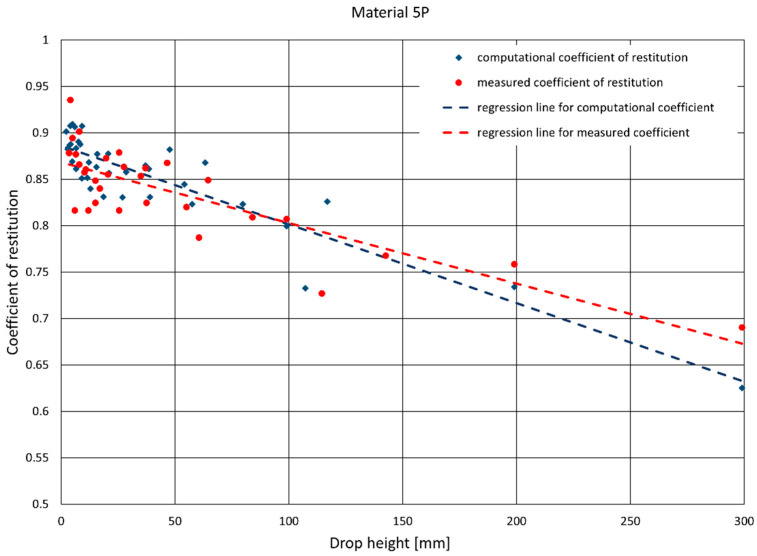
Sample graph of the set of values of the coefficient of restitution obtained in several consecutive attempts for one plate. The graph also shows straight lines of the linear regression for the measurement and computational values of the coefficient of restitution.

**Figure 12:**
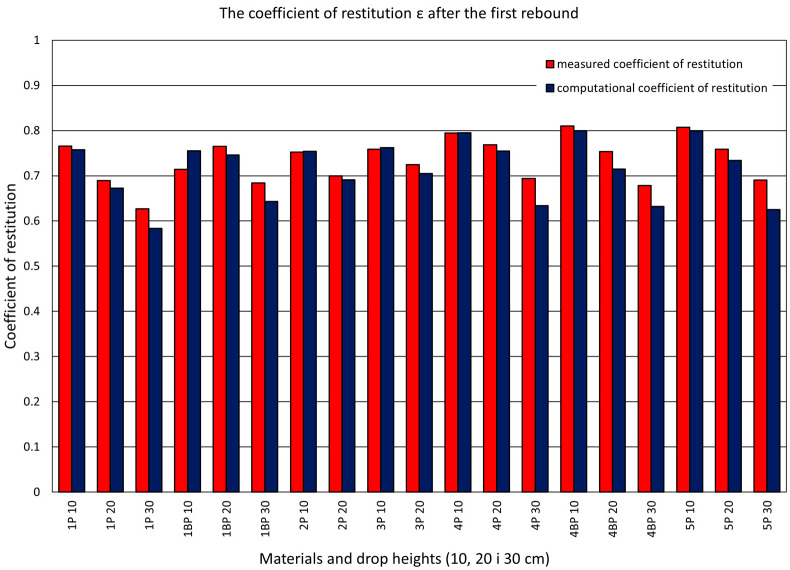
Comparison of the computational and measurement coefficients. The graph shows the values for the first rebound when falling from different heights (10—100 mm, 20—200 mm, 30—300 mm).

The values of the computational and measurement coefficients for one selected material, 5P, are presented on the graphs in Figure 6, Figure 7 and Figure 11. Figure 11 also features straight lines obtained by the linear regression method, representing the dependence of the coefficient of restitution on the ram’s drop height. Figure 12 presents complete results for the samples from the whole set. The data concern the values of the measurement and computational coefficients of restitution determined for the first rebound, taking into account the height of the ram’s free fall.

**Figure 13:**
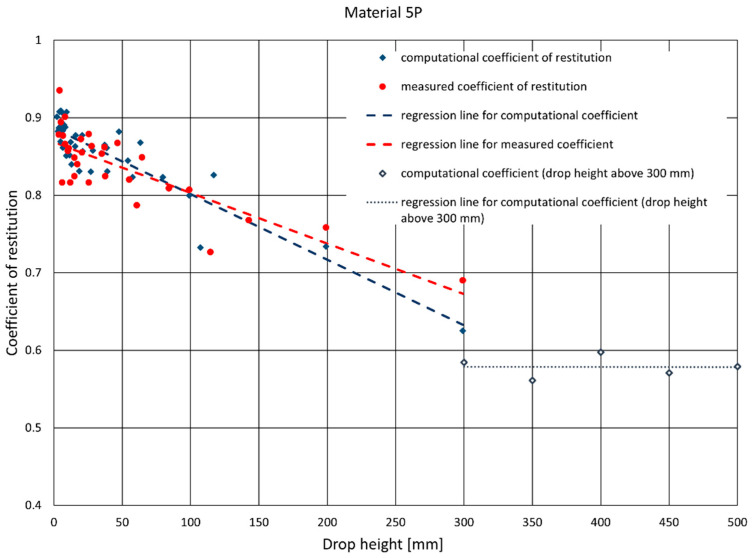
Graph of the set of values of the coefficient of restitution extended with the values of the computational coefficient of restitution obtained during drops from heights exceeding 300 mm—for these points, a separate regression line was determined.

In order to extend the data set obtained during drops from a height of up to 300 mm, subsequent experiments were carried out by letting the ram drop from larger heights—350, 400, 450, and 500 mm (Figure 13). These tests were carried out only using measurement equipment (acceleration and distance sensors), and no video recording was made. Therefore, only values of the computational coefficient of restitution were obtained. During the tests, some of the plates were damaged (4P, 4BP, and 5BP). Some materials (2BP and 3BP) had already shown signs of damage before these tests, which made the data obtained for their measurements inconsistent. Therefore, they have not been included in Table 3 and in the graph in Figure 12.

A record of physical quantities was obtained as a result of the study, which indirectly allowed for the determination of the coefficients of restitution of rectangular plates made of composites with hybrid reinforcement. Two algorithms were used to determine the coefficient: the first was measurement—the value of the coefficient was determined by measuring the height of the ram’s rebound, and the second was computational—the value of the coefficient was calculated by measuring the time elapsed between successive rebounds. After analyzing the obtained results of the measuring method, it was found that the value of the coefficient of restitution depended on the height from which the ram was dropped. As a result of testing the samples from the second set, it was confirmed that this dependence is close to linear, with a slight negative slope of the regression line. It can be seen that, for the computational coefficient, the slope angle of the regression line with respect to the ordinate axis is greater than that of the measurement coefficient. The results show that for larger drop heights (300 mm), the computational coefficient is smaller than the measurement coefficient, and for smaller heights (below 200 mm) the situation is reversed. At the same time, it was noticed that the coefficient of restitution for larger drop heights (over 300 mm) ceased to change significantly with an increase in the ram’s drop height.

Both methods of determining the coefficient of restitution provided similar results, and an analysis of the differences between them allowed for the identification of certain characteristic features of the computational method. It was noticed that the results obtained using this method are higher than results obtained using the measurement method in the scope of small drop heights of the ram, and for heights exceeding 300 mm they are lower. This is an important observation because the determination of the coefficient of restitution with the computational method is simpler than with the measurement method. It only requires the determination of the time between successive rebounds of the ram, based on any given measurement (in this case, it was the measurement of acceleration acting on the ram). The measurement method, on the other hand, requires an accurate measurement of the rebound height of the ram. The way in which this measurement was conducted in the study was very work-intensive and required the use of expensive additional equipment (a high-speed digital camera). By knowing the differences between the results obtained using both methods, we are able to estimate the possible results obtained by using one of the methods, on the basis of the results obtained with the other. In the future, we can obtain a reliable approximation of the value of the coefficient of restitution by only using one, simpler method.

During the tests, some plates were damaged. Minor cracks in the binders and single reinforcement strands were noticed. First of all, the damage was found in composites that included veneer layers. The wood, with a drop height exceeding 300 mm, was damaged due to a large deformation of the plate at the moment of the ram’s impact.

In the studies presented here, only one plate-hitting scenario was implemented. The ram of a given shape—the end of the ram hitting the plate was spherical—was dropped vertically onto a horizontally arranged flat sample. This certainly does not exhaust many other possibilities that may arise in reality. Expanding on knowledge about the hardness of composite materials, including the determination of the coefficient of restitution, requires further research which takes into account changes in the shape and size of the element hitting the sample, changes in the shape of the sample and the direction of impact. Problems of this kind occur especially in ballistic tests of various types of coverings protecting people and electronic and mechanical equipment. Such comprehensive studies, while apparently necessary, were beyond the scope of the work presented in this paper.

## 5. Conclusions

1. The values of the coefficient of restitution obtained using two described methods, due to slight differences (6% on average), can be considered acceptable. This means that both measurement methods can be used interchangeably, but the authors emphasize that the computational method is a simpler method that requires less expensive equipment.
2. The value of the coefficient of restitution changes as a function of the ram drop height. The greater the height, the smaller the value of the coefficient. In the authors’ opinion, this is so because, for greater heights more energy is dissipated during ever-greater deformations of the sample material. This is due to the micro-skid inside the composite structure. Such a situation will continue until the strength limit values are exceeded, resulting in the destruction of the sample.
3. The use of averaging over successive rebounds results in an increase (on average by 10–20% for the first three rebounds and by 20–25% for the first six rebounds for both methods) in the value of the restitution coefficient.
4. The use of vacuum laminating technology results in a slight increase in the value of the coefficient of restitution (except for material 3P/BP). It follows that with better resin supersaturation (higher fiber/resin ratio) there should be lower internal damping in the composite. However, it is the type of reinforcement used rather than the production method that mainly affects the value of the coefficient of restitution of tested materials. Finally, it should be remembered that decisions regarding whether a high or a low coefficient of restitution is required depends primarily on the final application of the composite.

## Figures and Tables

**Table 1:** Layers configuration and mechanical properties of tested materials.

Material Number	Layers Configuration	Area Density (g/m^2^) (Reinforcement by Mass)	Thickness (mm)	Young’s Modulus (GPa)	Tensile Strength (MPa)	Flexibility (mm/N)	Notes (Technology)
1 P	W + KW + W + KW	1032	0.9	41.3	346	0.015	Vacuum bag
1 BP	W + KW + W + KW	1725	1.4	35.6	301	0.012	No vacuum bag
2 P	W + S + W	816	0.7	35.1	343	0.021	Vacuum bag
2 BP	W + S + W	1279	1.0	27.6	251	0.02	No vacuum bag
3 P	KW + S + W	805	0.7	30.9	324	0.025	Vacuum bag
3 BP	KW + S + W	1273	1.0	24.1	258	0.019	No vacuum bag
4 P	W + F + KW	986	1.1	19.9	167	0.018	vacuum bag
4 BP	W + F + KW	1337	1.3	15.6	150	0.014	no vacuum bag
5 P	F + S + W + KW	1240	1.3	24.1	214	0.023	vacuum bag
5 BP	F + S + W + KW	1708	1.5	17.3	175	0.017	no vacuum bag

**Table 2:** Materials used for reinforcement of tested composites.

Layer	Material	Grammage (g/m^2^)
F	Wood veneer	343
KW	Woven kevlar–carbon fabric (plain hybrid)	165
S	Woven glass fabric (plain weave)	200
W	Woven carbon fabric (plain weave)	204
In all cases the epoxy resin (EPOLAM 2017) was used as a matrix		

**Table 3:** Linear regression parameters of the dependence *ε*(*h*).

Material	Coefficient of Restitution	Slope *a*	Intercept *b*	Correlation Coefficient	Standard Deviation of the Approximation
1P	computational	−0.0011	0.8810	0.9432	0.0226
	measurement	−0.0008	0.8383	0.9520	0.0175
1BP	computational	−0.0009	0.8835	0.8871	0.0284
	measurement	−0.0007	0.8624	0.8028	0.0358
2P	computational	−0.0011	0.8895	0.9501	0.0164
	measurement	−0.0007	0.8401	0.7924	0.0270
3P	computational	−0.0012	0.9131	0.8649	0.0332
	measurement	−0.0007	0.8533	0.8128	0.0260
4P	computational	−0.0007	0.8819	0.9415	0.0154
	measurement	−0.0006	0.8721	0.9144	0.0178
4BP	computational	−0.0006	0.8850	0.7903	0.0202
	measurement	−0.0006	0.8752	0.7743	0.0227
5P	computational	−0.0008	0.8861	0.9126	0.0227
	measurement	−0.0007	0.8683	0.8267	0.0294

## Data Availability

Data is contained within the article.
